# Hemophagocytic lymphohistiocytosis secondary to virus infection and followed by lupus nephritis recurrence in a renal transplantation pediatric recipient: a case report

**DOI:** 10.1186/s12882-023-03249-4

**Published:** 2023-07-03

**Authors:** Jiyuan Li, Chen Gao, Xuejing Zhu, Danyi Yang, Wendan Mao, Hengchang Yao, Mingyang Deng, Liang Tan, Helong Dai, Xubiao Xie, Longkai Peng, Fenghua Peng

**Affiliations:** 1 Department of Kidney Transplantation, Center of Organ Transplantation, The Second Xiangya Hospital of Central South University, Changsha, China; 2 Department of Nephrology, The Second Xiangya Hospital, Central South University, Changsha, Hunan China; 3 Hunan Key Laboratory of Kidney Disease and Blood Purification, Changsha, Hunan China; 4 Department of Hematology, The Second Xiangya Hospital of Central South University, Changsha, China; 5 Clinical Research Center for Organ Transplantation in Hunan Province, Changsha, China; 6 Clinical Immunology Center, Central South University, Changsha, China

**Keywords:** Hemophagocytic lymphohistiocytosis (HLH), Renal transplant, Virus infection, Systemic lupus erythematosus (SLE), Lupus nephritis (LN)

## Abstract

**Background:**

Hemophagocytic lymphohistiocytosis (HLH) is a rare and life-threatening disorder characterized by systemic inflammation and organ failure as a result of dysregulated immune cell activation. HLH can be induced by a variety of factors including infection, tumours and autoimmune disease and can also occur in patients following solid organ transplantation. Occurrence of HLH and lupus nephritis (LN) successively within a short period of time after renal transplantation is uncommon.

**Case presentation:**

We described an 11-year-old female post-transplant patient who presented with hemocytopenia, fever, elevated serum ferritin, splenomegaly, hyperlipidemia, and hypofibrinemia, and was clinically diagnosed with HLH. After comprehensive treatment with corticosteroids, intravenous immunoglobulin (IVIG), and reducing immunosuppressants, her condition improved, but then hematuria ensued. The transplant kidney biopsy showed LN. She was treated with hydroxychloroquine and methylprednisolone while intensive immunosuppressive agents were given. She has remained in remission for two years until now.

**Conclusions:**

The main inducing factors of HLH should be identified as early as possible, and accurate treatment plans should be taken. The long-course IVIG regimen may be one of the effective treatments for virus-induced HLH. After remission of HLH, we need to be alert to the recurrence of autoimmune diseases in patients with underlying diseases, and timely increase immunosuppressants.

**Supplementary Information:**

The online version contains supplementary material available at 10.1186/s12882-023-03249-4.

## Background

Hemophagocytic lymphohistiocytosis (HLH), also known as hemophagocytic syndrome, is a rare and fatal disease. In East Asian populations, the prevalence is approximately 1 in 800,000 [1], and in kidney transplant recipients, it is approximately 0.4% [2]. HLH is characterized by the dysregulated activation of cytotoxic T lymphocytes, natural killer (NK) cells, and macrophages, resulting in systemic inflammatory symptoms and multi-organ system damage. HLH can be divided into two categories: primary and secondary. Primary HLH is mostly seen in children, and it is an autosomal recessive inheritance or X-chromosome-linked inheritance disease caused by the existence of HLH-related gene defects. Secondary HLH is most common in adults around 50 years of age and is caused by etiological stimuli such as infection (42%), malignancy (40%), or autoimmune diseases (11%), and it can also be induced by hematopoietic stem cell transplantation and solid organ transplantation [3, 4]. In renal transplant patients, the prognosis is poor, and mortality can be as high as (47–53)% [2, 5]. In our report, we described a case of HLH secondary to virus infection and followed by lupus nephritis (LN) recurrence in a renal transplantation pediatric recipient.

## Case presentation

**Fig. 1:**
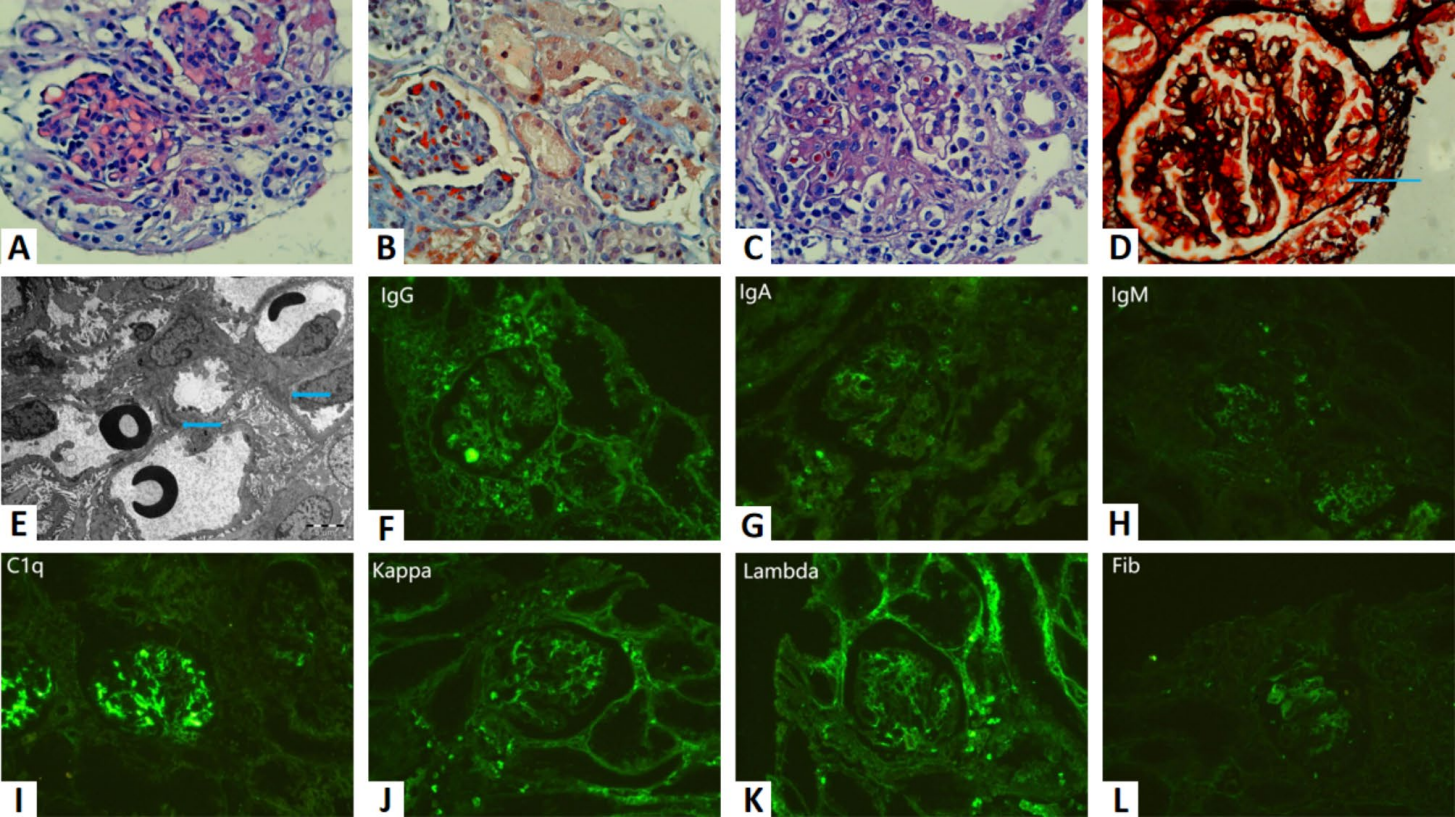
Pathological findings. **(A, B)** Preimplantation biopsy shows glomerular microthrombosis (HE, Masson, respectively). (C-L)Transplant kidney biopsy: **(C)** Light microscopy shows crescentic glomerulonephritis (HE). **(D)** Glomerular basement membrane rupture (long arrow), blood cell and fibrin extravasation (PASM). **(E)** Dense deposit in the mesangial and paramesangial regions on EM (short arrow). **(F-L)** Immunofluorescence shows that three kinds of antibodies IgG, IgM, IgA, one kind of complement C1q, two kinds of light chain Kappa, Lambda, and Fibrin are positive, while complement C3 is negative (no exhibition)

On August 14, 2020, an 11-year-old female patient was admitted with symptoms of anemia and leukopenia eight months after a renal transplant. A cadaveric donor renal transplant was performed on July 6, 2020. The donor was a two-year-old male who suffered brain death. The pre-implantation biopsy of the donor graft showed focal microthrombi in the glomerulus (Fig. 1). Induction with anti-thymocyte globulin, methylprednisolone, and triple maintenance immunotherapy including tacrolimus, mycophenolate mofetil (MMF) and methylprednisolone were applied. Postoperatively, the transplanted kidney functioned well. She was discharged on July 30, 2020, with creatinine 90.7 umol/L, hemoglobin 87 g/L, white blood cell 6.34 × 10^9^/L, and platelet 132 × 10^9^/L.

Past medical history: the patient was diagnosed with uremia due to LN at age ten. Laboratory tests showed: creatinine 708 umol/L, ANA 1:640 (+), and dsDNA (+). However, no renal biopsy was performed.

The patient presented a three-stage clinical course following admission as below.

**Fig. 2:**
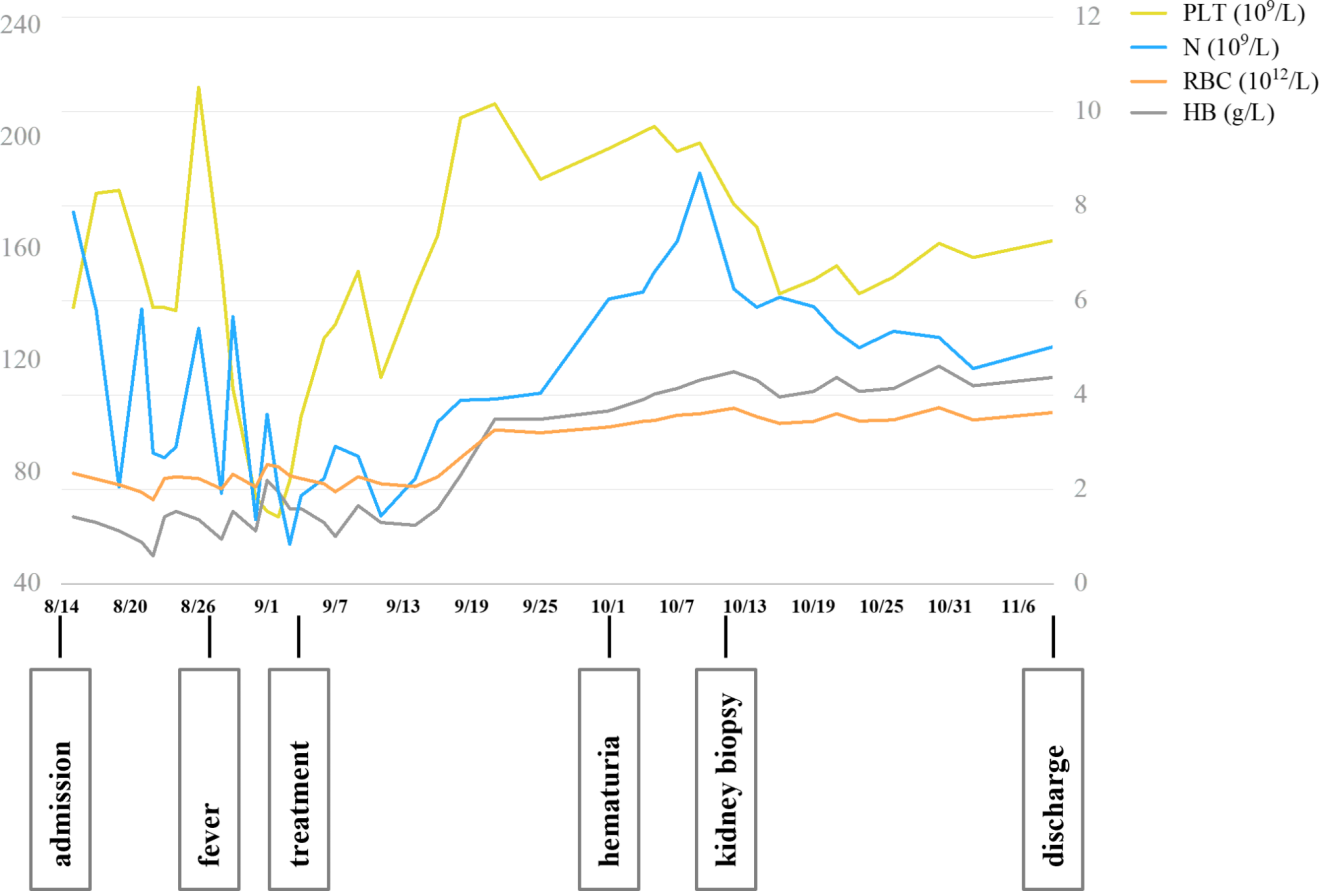
Schematic diagram of the patient’s blood cells changes and clinical course after admission. Despite a series of symptomatic treatment after admission, the patient’s red blood cells, neutrophils and platelets repeatedly decreased rapidly. On September 4, hemocytopenia started to improve after comprehensive treatment. Note: The PLT and HB lines are drawn according to the left scale, and the N and RBC lines are drawn according to the right scale. PLT, platelets; N, neutrophils; RBC, red blood cells; HB, hemoglobin

Stage 1 (Anemia): On August 14, the patient was admitted with fatigue and laboratory data showed anemia (hemoglobin: 72 g/L) (Table 1). MMF decreased from 0.75 g/d to 0.5 g/d, recombinant human granulocyte stimulating factor and erythropoietin were infused. From August 22 to September 7, she received infusions of leukocyte-free erythrocyte suspension at 1.0–1.5u/per time for four times and immunoglobulin 5 g/d for ten days, but she still suffered from further pancytopenia without any significant improvement (Fig. 2).

**Table 1:** Relevant laboratory findings

	Stage1 (Anemia)	Stage2 (Fever)	Stage3 (Hematuria)	Discharge	Follow-up	Normal range
**Complete blood cell count**						
WBC count (10^9^/L)	3.71	2.91	8.53	7.63	8.31	5–12
Absolute neutrophil count (10^9^/L)	2.15	1.92	6.03	5.02	4.54	1.8–6.3
Hemoglobin (g/l)	72	56	102	114	111	110–160
Platelet count (10^9^/L)	152	153	196	168	230	100–400
**Chemistry**						
Creatinine (umol/l)	68	66.2	78	65	80	44–133
BUN (mmol/l)	9.48	3.82	21	19.6	5.7	2.9–7.14
Uric acid (umol/l)	239.7	279	334	330	187	155–357
**Urinary analysis**						
protein	Negative	Negative	+		Negative	
Leukocyte esterase	Negative	Negative	Negative		Negative	
Glucose	Negative	Negative	+-		+-	
Occult blood	Negative	Negative	+++		+	
RBC (/HP)	0	0	+++			0
WBC (/HP)	0	0–1	0			0–3
24-h urine protein (mg/day)			1573.11			0-150
Urine culture			*Proteus mirabilis*	Negative		
**Immune serologies**						
C1q		2.3	5.32			<10
ANA (1: 80)		Positive	Negative			
ANA (1: 160)		Positive	Negative			
ANA (1: 320)		Negative	Negative			
Anti-dsDNA		Positive	Negative			
Anti-SM		Negative				
C3 (g/l)		0.55	0.46			0.79–1.52
C4 (g/l)		0.18	0.12			0.16–0.38
ESR (mm/h)		9				0–20
**Anemia-related tests**						
Serum vitamin B12 (pg/ml)	554					187–883
Olic acid (ng/ml)	>20					3.1–20.5
Thalassemia gene test	Negative					
Sucrose hemolysis test	Negative					
Direct anti-human globulin (IgG) test	Negative					
Direct anti-human globulin (C3) test	Negative					
TRF (g/l)		1.03				2.02–3.36
**Infectious tests**						
CRP (mg/l)		2.07				0–8
PCT (ng/ml)		0.126	0.058			0-0.05
G text		78.5	<37.5			<70
GM text		0.02				<0.5
Blood culture		Negative				
Bone marrow culture		Negative				
**Virus DNA quantification**						
CMV-DNA (copies/ml)	Negative	Negative	142			<500
B19-DNA (ct)	7.21	4.57	31.3			>38
JCV-DNA (copies/ml)	Negative					<2.0E + 03
BKV-DNA (copies/ml)	Negative		Negative			<2.0E + 03
EBV-DNA(copies/ml)	22.66	1.27E + 03	Negative	393.2		<500
EBV-DNA in B cells (copies/105cells)		18.21				
EBV-DNA in T cells (copies/105cells)		45.3				
EBV-DNA in NK cells (copies/105cells)		Negative				
**HLH-specific tests**						
Fibrinogen (g/l)		1.94	1.76			2–4
Triglyceride (mmol/l)		1.91	0.73			<1.71
Ferritin (ng/l)	332.9	622.9	99.71			4.63–204
Soluble interleukin-2 receptor (u/ml)		2903				223–710
Natural killer cell activity (pg/ml)		2.55%				
Genetic testing for HLH		Negative				

WBC, white blood cell; BUN, blood urea nitrogen; RBC, red blood cell; C1q, complement component 1q; ANA, antinuclear antibody; Anti-dsDNA, anti-double stranded deoxyribonucleic acid; C3, complement 3; C4, complement 4; ESR, erythrocyte sedimentation Rate; TRF, transferrin; CRP, c-reactive protein; PCT, procalcitonin; GM, glactomannan; DNA, deoxyribonucleic acid; JCV, John Cunningham virus; BKV, BK virus; EBV, Epstein-Barr virus; NK, natural killer; HLH, hemophagocytic lymphohistiocytosis

**Fig. 3:**
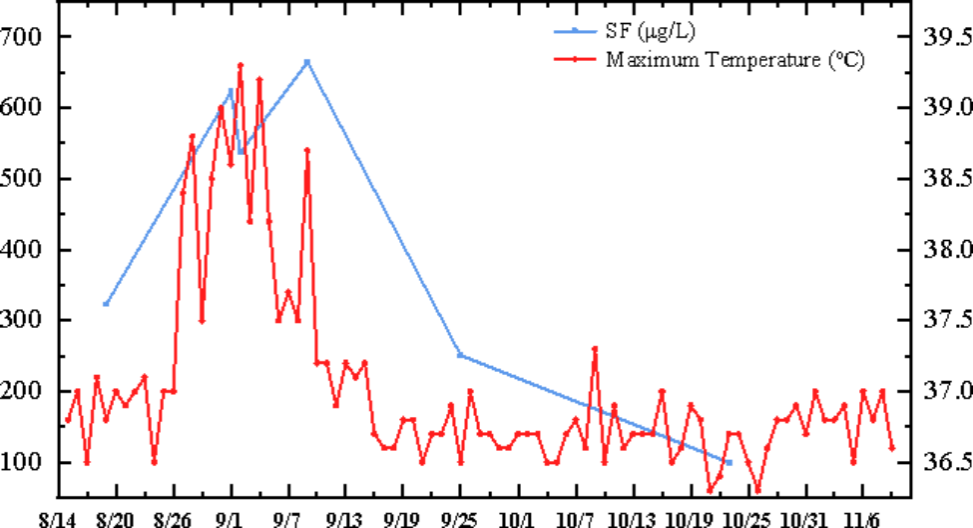
Ferritin and maximum body temperature variation graph

Stage 2 (Fever). On the morning of August 26, she had a sudden onset pain of bilateral knee and sacroiliac joint, which was completely relieved by bucinnazine. On the next day, she started to develop remittent fever, fluctuating between 36.0℃ and 39.3℃ without chills or cough (Fig. 3). The relevant serological indicators (Table 1) of bacteria and fungi were negative, and the condition did not improve with antibiotic treatment. The histology of bone marrow aspiration revealed the normal hematopoietic elements, and no evidence of phagocytosis. Computed tomography showed mild splenomegaly. According to the systemic lupus erythematosus disease activity index (SLEDAI), the total score in this patient was three and was considered as basically inactive lupus. However, according to the HLH-2004 guidelines (Table 2) [6], the patient met four of the eight indicators for the diagnosis of HLH: (i) fever > 38.5℃; ii) splenomegaly; iii) hemocytopenia; and iv) elevated serum ferritin. She was tentatively diagnosed with HLH, based on a HScore score of 182 [7]. Soluble CD25 and NK cell activity were later reported to be fit for the criterion. Hence, HLH was confirmed. Genetic tests showed no pathogenic mutation. Several viruses were active including EBV, B19 virus, and CMV. EBV sorting was tested to guide treatment (Table 1). After establishing the initial diagnosis of HLH on September 4, we stopped MMF, converting tacrolimus to cyclosporine (C0: 50–100ng/mL, C2: 400–500ng/mL), and prescribed hydroxychloroquine 75 mg, Q12h (5 mg/kg/d) to treat the underlying disease of systemic lupus erythematosus (SLE). Immunoglobulin 0.4 g/kg/d was given for 3 days then 0.15 g/kg/d for 15 days; methylprednisolone 40 mg/d for 5 days, thereafter tapered to methylprednisolone at a dose of 8 mg/d for four weeks. After treatment, patient’s condition improved, MMF restarted with 0.25 g daily.

**Table 2:** Diagnosis of HLH

One of the two conditions is met	
(a) Pathological mutations are found in the HLH-related pathogenic genes.	
(b) At least five of the following eight criteria are met.	
1. Fever: temperature > 38.5℃.	
2. Hemocytopenia: hemoglobin < 90 g/L, platelets < 100*10^9^/L, neutrophils < 1.0*10^9^/L and not due to bone marrow hypoplasia.	
3. Splenomegaly.	
4. Hypertriglyceridemia > 3mmol/L or hypofibrinogenemia < 1.5 g/L.	
5. Phagocytes found in bone marrow, spleen or lymph nodes.	
6. Serum ferritin ≥ 500 µg/L.	
7. Decreased or absent NK cell activity.	
8. Elevated soluble interleukin-2 receptor (sCD25) level ≥ 2400µ/ml.	

HLH, Hemophagocytic lymphohistiocytosis

Stage 3 (Hematuria). On October 1, the patient developed gross hematuria with no fever, no lumbar pain, and no urinary irritation. The urine bacterial culture (Table 1) was suggestive of *proteus mirabilis*, and no improvement was observed after anti-infective treatment according to the bacterial susceptibility testing. On October 12, a transplant kidney biopsy was performed, and the pathological diagnosis was LN (NIH classification: type IIIa, NIH lupus nephritis (LN) score AI 6, CI 0, Fig. 1). Oral hydroxychloroquine was continued at 75 mg every 12 h; methylprednisolone was increased to 16 mg per day; cyclosporine was increased to guarantee C0 > 200ng/mL, and MMF was added to sufficient dosage, keeping the MPA-AUC 0–12 h at 50–60ug·h/mL. Her condition improved and she was discharged on November 9.

After discharge, the patient was admitted four times for the asymptomatic urinary infections, which were relieved after anti-infection treatment. Currently, she has been followed up for over two years with stable condition (Table 1).

## Discussion and conclusions

The diagnosis of HLH is usually based on the HLH-2004 guidelines [6]. In this case, the patient was found to have hemocytopenia, fever, splenomegaly, and elevated serum ferritin, while did not fully met the diagnostic criteria. Therefore, we further performed the complex and time-consuming assays for NK cell activity and sCD25 level to confirm the diagnosis. Meanwhile, we used a simplified diagnostic procedure for the initial diagnosis of HLH, which calculated a HScore score of 182 and indicated a 70% probability of HLH [7]. This patient had obvious predisposing factors: post-renal transplantation, EBV, B19 virus, and a history of LN, with no abnormalities in genetic testing, thus it was more likely to be secondary HLH. Based on the SLEDAI score which showed that lupus was largely inactive at that time, and given the clear correlation between EBV copy number and HLH progression, we considered that EBV played a major role in this case.

Treatment of non-transplant HLH patients is also primarily followed the HLH-2004 regimen [6], which recommends the use of high-dose steroids in combination with the topoisomerase-II inhibitor etoposide. In addition, IVIG or cyclosporine is appropriate in some cases. However, in renal transplant patients, etoposide is usually unsuitable due to high risk of death [5]. If the infection is induced by viruses such as EBV, CMV, or B19 virus, the treatment will generally begin with a reduction of immunosuppression. Meanwhile short-term (usually about 3 days) and high-dose regimen (accumulated dose of 1.6-2 g/kg body weight) are mostly used to control the virus in a short period of time [8–10], Nevertheless, several cases of death due to recurrence have been reported [5]. According to our experience and literature review, multiple viruses, especially B19 virus, were not so easy to be controlled in such a short term (less than 7 days) in transplant cohort [11]. So we modified this regimen into a long-course (IVIG 0.15-0.4 g/kg/d, > 15 days) scheme to gradually rebuild and restore the patient’s immunity. EBV infection with different lymphocytes such as T-cell, B-cell or NK cells will have different clinical manifestations, prognosis, and treatment plans [12], therefore EBV sorting PCR was introduced to guide the treatment. For EBV-HLH with only or mainly B-lymphocyte infection, rituximab is an effective treatment [13], nevertheless, this patient did not fit the criteria apparently.

It was particularly rare for post-transplant patients to develop LN immediately after HLH relief. A study based on the united network for organ sharing (UNOS) data showed the recurrence rate of LN after renal transplantation was 2.44% [14], however, some studies reported recurrence in 30-44% of recipients using a complete histologic examination of biopsies [15, 16]. Although a study displayed a close relationship between SLE and HLH [17], we did not find any case report about LN recurrence just following HLH relief in transplant patients yet. In this case, LN may partially attributed to insufficient immunosuppression, as we completely stopped MMF and adopted a low dose of cyclosporine for a long time to control HLH.

It is noteworthy that about half of LN recurrence patients may develop rejection. Meanwhile, HLH may exhibit kidney thrombotic microangiopathy and further complicate the diagnosis [8, 18]. Therefore, the importance of kidney biopsy needs to be emphasized. Fortunately, the pathology in this case confirmed only a simple LN, making the treatment relatively simple.

Conclusively, HLH should be considered for renal transplant patients with fever and unexplained obstinate hemocytopenia. The main causation should be identified as early as possible. When hematuria and proteinuria of unknown etiology occur after HLH treatment, especially when the patient has a past history of autoimmune disease, it is recommended to perform renal transplant biopsy. The treatment of virus induced HLH and LN after renal transplantation is slightly different from the guidelines for non-transplanted patients, we believe that this case can provide an option for the treatment of HLH and LN recurrence after renal transplantation in children.

## Electronic supplementary material

Below is the link to the electronic supplementary material.

Supplementary Material 1

## Data Availability

The datasets analysed during the current study are available from the corresponding author on reasonable request.

## Declarations

**Ethics approval and consent to participate**.

The patient and her parents were informed about the availability and importance of the data, including the clinical data, images, and health information, described in this article.

**Consent for publication**.

We have obtained informed consent in writing from the patient and her parents.

### Authors details

^1^Department of Kidney Transplantation, Center of Organ Transplantation, The Second Xiangya Hospital of Central South University, Changsha, China; ^2^Department of Nephrology, The Second Xiangya Hospital, Central South University, Changsha, Hunan, China; ^3^Hunan Key Laboratory of Kidney Disease and Blood Purification, Changsha, Hunan, China; ^4^Department of Hematology, The Second Xiangya Hospital of Central South University, Changsha, China; ^5^Clinical Research Center for Organ Transplantation in Hunan Province, Changsha, China; and ^6^Clinical Immunology Center, Central South University, Changsha, China.

## Abbreviations

HLH: hemophagocytic lymphohistiocytosis
LN: lupus nephritis
IVIG: intravenous immunoglobulin
NK: natural killer
MAS: macrophage activation syndrome
MMF: mycophenolate mofetil
SLEDAI: systemic lupus erythematosus disease activity index
SLE: systemic lupus erythematosus
UNOS: united network for organ sharing

## References

[1] Hayden A, Park S, Giustini D, Lee AYY, Chen LYC (2016). Hemophagocytic syndromes (HPSs) including hemophagocytic lymphohistiocytosis (HLH) in adults: a systematic scoping review. Blood Rev.

[2] Ponticelli C, Alberighi ODC (2009). Haemophagocytic syndrome–a life-threatening complication of renal transplantation. Nephrol Dial Transplant.

[3] Campo M, Berliner N (2015). Hemophagocytic lymphohistiocytosis in adults. Hematol Oncol Clin North Am.

[4] Smits BM, van Montfrans J, Merrill SA, van de Corput L, van Gijn M, de Vries A, van den Bos C, Abbink F, van der Molen RG, Dors N (2021). A minimal parameter set facilitating early decision-making in the diagnosis of Hemophagocytic Lymphohistiocytosis. J Clin Immunol.

[5] Valdés Francí E, Perez Flores I, Candel FJ, de la Moreno MA, Romero NC, Rodríguez Cubillo B, Lucena Valverde R, Sánchez Fructuoso AI (2021). Hemophagocytic syndrome triggered by donor-transmitted toxoplasmosis as a complication in same-donor recipients of renal transplantation: case report and review of the literature. Transpl Infect Dis.

[6] Henter J-I, Horne A, Aricó M, Egeler RM, Filipovich AH, Imashuku S, Ladisch S, McClain K, Webb D, Winiarski J (2007). HLH-2004: Diagnostic and therapeutic guidelines for hemophagocytic lymphohistiocytosis. Pediatr Blood Cancer.

[7] Fardet L, Galicier L, Lambotte O, Marzac C, Aumont C, Chahwan D, Coppo P, Hejblum G (2014). Development and validation of the HScore, a score for the diagnosis of reactive hemophagocytic syndrome. Arthritis Rheumatol.

[8] Steffen CJ, Koch N, Eckardt KU, Amann K, Seelow E, Schreiber A (2021). Hemophagocytic lymphohistiocytosis and thrombotic microangiopathy after parvovirus B19 infection and renal transplantation: a case report. BMC Nephrol.

[9] Singh NS, Pagano AL, Hays AJ, Kats A, Dahl SM, Warady BA, Beins NT, Yin DE (2022). Ehrlichia-induced hemophagocytic lymphohistiocytosis in a pediatric kidney transplant recipient. Pediatr Transpl.

[10] Naciso Júnior J, Neri BdO, Dantas GLdA (2020). Silveira LdHJ, sales MLdMBO, Freitas TVdS, Esmeraldo RdM: secondary hemophagocytic syndrome after renal transplantation: two case-reports. Jornal Brasileiro de Nefrologia.

[11] Bentata Y (2021). Parvovirus B19 in kidney transplantation: key points and essential pitfalls to know. Infect Dis (Lond).

[12] Zhang P, Zeng C, Cheng J, Zhou J, Gu J, Mao X, Zhang W, Cao Y, Luo H, Xu B (2019). Determination of Epstein-Barr Virus-Infected lymphocyte cell types in Peripheral Blood mononuclear cells as a Valuable Diagnostic Tool in Hematological Diseases. Open Forum Infect Dis.

[13] Meng G-Q, Wang J-S, Wang Y-N, Wei N, Wang Z (2021). Rituximab-containing immuno-chemotherapy regimens are effective for the elimination of EBV for EBV-HLH with only and mainly B lymphocytes of EBV infection. Int Immunopharmacol.

[14] Contreras G, Mattiazzi A, Guerra G, Ortega LM, Tozman EC, Li H, Tamariz L, Carvalho C, Kupin W, Ladino M (2010). Recurrence of lupus nephritis after kidney transplantation. J Am Soc Nephrology: JASN.

[15] Goral S, Ynares C, Shappell SB, Snyder S, Feurer ID, Kazancioglu R, Fogo AB, Helderman JH (2003). Recurrent lupus nephritis in renal transplant recipients revisited: it is not rare. Transplantation.

[16] Nyberg G, Blohmé I, Persson H, Olausson M, Svalander C (1992). Recurrence of SLE in transplanted kidneys: a follow-up transplant biopsy study. Nephrol Dial Transplant.

[17] Kim J-M, Kwok S-K, Ju JH, Kim H-Y, Park S-H (2012). Reactive hemophagocytic syndrome in adult korean patients with systemic lupus erythematosus: a case-control study and literature review. J Rheumatol.

[18] Bae MN, Kwak DH, Park SJ, Choi BS, Park CW, Choi YJ, Lee JW, Yang CW, Kim Y-S, Chung BH (2016). Acute kidney injury induced by thrombotic microangiopathy in a patient with hemophagocytic lymphohistiocytosis. BMC Nephrol.

